# Sensory Electrical Stimulation Cueing May Reduce Freezing of Gait Episodes in Parkinson's Disease

**DOI:** 10.1155/2018/4684925

**Published:** 2018-08-01

**Authors:** Lois Rosenthal, Dean Sweeney, Anne-Louise Cunnington, Leo R. Quinlan, Gearóid ÓLaighin

**Affiliations:** ^1^Rehabilitation and Movement Disorder, Department for Care of Elderly, Stobhill Hospital, Glasgow, UK; ^2^Electrical & Electronic Engineering, School of Engineering and Informatics, NUI Galway, University Road, Galway, Ireland; ^3^Human Movement Laboratory, CÚRAM Centre for Research in Medical Devices, NUI Galway, University Road, Galway, Ireland; ^4^Department of Cardiovascular and Medical Sciences, University of Glasgow, Glasgow, UK; ^5^Physiology, School of Medicine, NUI Galway, University Road, Galway, Ireland

## Abstract

**Introduction:**

Freezing of gait (FoG) is a movement abnormality that presents with advancing Parkinson's disease (PD) and is one of the most debilitating symptoms of the disease. The mainstay of nonpharmacological management of FoG is typically through external cueing techniques designed to relieve or prevent the freezing episode. Previous work shows that electrical stimulation may prove useful as a gait guidance technique, but further evidence is required. The main objective of this study was to determine whether a “fixed” rhythmic sensory electrical stimulation (sES) cueing strategy would significantly (i) reduce the time taken to complete a walking task and (ii) reduce the number of FoG episodes occurring when performing the task.

**Methods:**

9 participants with idiopathic PD performed a self-identified walking task during both control (no cue) and cueing conditions. The self-identified walking task was a home-based daily walking activity, which was known to result in FoG for that person. A trained physiotherapist recorded the time taken to complete the walking task and the number of FoG episodes which occurred during the task. Data were analyzed by paired *t*-tests for both the time to complete a walking task and the number of FoG episodes occurring.

**Results:**

sES cueing resulted in a reduction in the time taken to complete a walking task and in the number of FoG episodes occurring during performance of this task by 14.23 ± 11.15% (*p*=0.009) and 58.28 ± 33.89% (*p*=0.002), respectively.

**Conclusions:**

This study shows a positive effect of “fixed” rhythmic sES on the time taken to complete a walking task and on the number of FoG episodes occurring during the task. Our results provide evidence that sES cueing delivered in a “fixed” rhythmic manner has the potential to be an effective cueing mechanism for FoG prevention.

## 1. Introduction

Parkinson's disease (PD) is the second most common neurodegenerative disease in the developed world. There are an estimated 1.2 million people living with PD in the EU [1]. The disease is typically characterized by movement abnormalities that develop with the progression of PD. Freezing of gait (FoG) is a movement abnormality that presents in more advanced stages of the disease and is one of the most debilitating symptoms of PD. The unpredictable nature of FoG leads to increased falls risk and increased fear of falling for those affected, which can subsequently lead to additional nonmotor complications such as social isolation and depression [2].

The management of FoG is widely perceived by clinicians as a challenging task [3–5]. The most challenging type of FoG is on-state FoG, which refers to the case when people with Parkinson's (PwP) experience FoG while they are getting the full benefits of their dopaminergic medication. On-state FoG can occur either as a resistance to the otherwise positive effects of dopaminergic medication or in some cases, it can in fact be induced by the dopaminergic medication itself, which is otherwise having a beneficial effect for the PwP. These causes of on-state FoG make it particularly complex to manage through pharmacological interventions [3]. The current nonpharmacological mainstay technique for the management of FoG is the adoption of external cueing. External cueing techniques use forms of sensory stimuli to prevent or relieve the FoG episode, which may include the use of visual or auditory cueing devices. Examples of visual cueing devices are laser pointer canes [6] and visual cue glasses [7]. Examples of auditory cueing devices are discrete metronome devices [8] or smartphone-based metronome apps [9]. These different cueing techniques have varied levels of effectiveness, with inconclusive results frequently reported [10–13].

A limited number of studies have investigated alternative external cueing techniques, which can be easily concealed and adopted into routine daily practice [14, 15]. One such technique is electrical stimulation (ES) cueing. Studies have indicated that both motor (ES at an intensity sufficient to activate muscle contraction) and sensory (ES at an intensity sufficient to activate a sensory, but not motor response) ES cueing may prove useful [14, 15].

In these two studies, cueing was delivered using an “adaptive” rhythmic cueing strategy, where the delivery of cueing was synchronized to the participant's gait cycle in real time. Although this method of ES delivery is used extensively for the correction of drop foot, there is no significant evidence that adopting such a method will provide an optimum solution for reducing FoG. It has been reported that improvements in FoG severity in PD occur when auditory cueing is delivered using a “fixed” rhythmic cueing strategy [8]. During “fixed” rhythmic cueing, the rhythm is set at a fixed value based on the person's typical gait cycle. Various rhythm frequencies for “fixed” rhythmic cueing have been previously evaluated. These range from frequencies (i) equal to the person's typical step rate [13, 16], (ii) set to 10–20% below the person's typical step rate [8, 17, 18], (iii) set to 10–20% above the person's typical step rate [16, 19–21], or (iv) set to a perceived comfortable value [22]. Although the optimal frequency is unclear, it has been suggested that “fixed” rhythmic sensory stimuli help the PwP to synchronize stepping, thus helping them to regulate their gait rhythm.

Although the optimum cueing approach for sES is unclear, we hypothesize that a sES cueing strategy which provides a “fixed” rhythmic cue to synchronize stepping will provide enhanced cueing to reduce FoG and enhance walking speed. In this study, we assess if a sES cueing technique, which uses “fixed” rhythmic cueing, can reduce FoG for PwP in a home environment. The primary outcome measures were (i) the time taken to complete a walking task and (ii) the number of FoG episodes occurring during the execution of this task. A secondary outcome measure was the PwP's perceived comfort/pain level with sES cueing during walking tasks.

## 2. Methods

### 2.1. Participants

Nine (6 men and 3 women; mean age 73.22 ± 11.8 years; mean disease duration 13.33 ± 16.51; mean FoGQ score 13.89 ± 2.92; mode modified Hoehn and Yahr stage 2 (range: 1–3)) participants with idiopathic PD enrolled in the study. Participants were recruited through the Movement Disorder Clinic, Stobhill Day Hospital, Glasgow, Scotland, UK. All participants were informed about the nature of the study and provided written informed consent. The protocol was approved by the West of Scotland Research Ethics Service. Participants upon clinical assessment reported experiencing FoG despite taking their medication and thus were not required to abstain from their medication during participation in the study. At recruitment, the severity of the disease was assessed using the modified Hoehn and Yahr stage score, and the participants' global cognitive function was assessed using the Mini-Mental State Examination (MMSE). Inclusion criteria for the protocol were a modified Hoehn and Yahr stage score of 1–4, MMSE score greater than 24, and a history of FoG.

### 2.2. sES Cueing Technique

**Figure 1:**
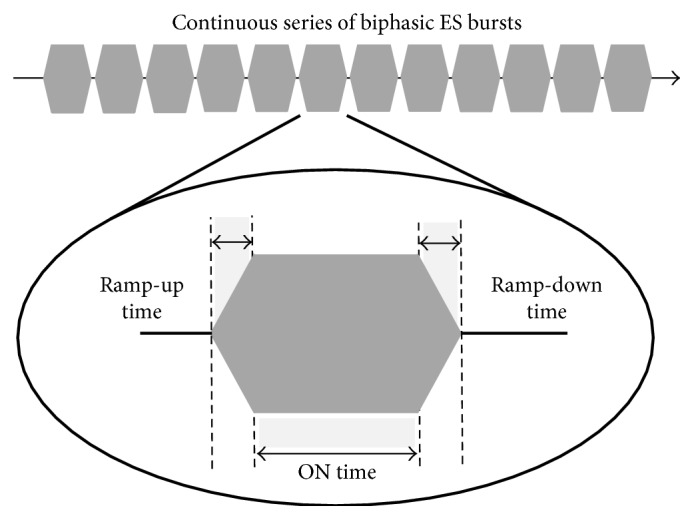
Utilized sES rhythmic cueing strategy.

Cueing was delivered using a custom-built electrical stimulator, cueStim. The cueStim device is a voltage-controlled two-channel stimulator. The device is worn on the waist and is controlled wirelessly allowing the participant to walk freely during the test. During sES cueing, the fixed rhythmic cueing strategy delivered a continuous series of biphasic ES bursts. Each burst consisted of 100 ms ramp-up time, 500 ms ON time, 100 ms ramp-down time, and 0 ms OFF time, Figure 1. The amplitude of the stimulation burst was adjusted for each participant such that a sensory response was elicited but that the amplitude was not of sufficient intensity to produce a motor response. The delivery of the sES cueing was facilitated through the use of 5 × 5 cm skin surface electrodes (Nidd Valley Medical Limited, England) placed over the motor points of the hamstring or quadriceps muscle of the body side most affected by PD.

### 2.3. Experimental Protocol

All assessment was carried out during the medicated “on-state” of the participant (self-reported by the participant). Each participant identified a walking task within their home, which usually elicited FoG episodes, Table 1. The common features in each of the walking tasks performed were that each walking task included performing a turn during walking, walking through a doorway/doorways, walking across a room, and walking in a corridor/hallway. The participant performed the task twice, once with sES cueing (cueing) and once without sES cueing (control). The order of test condition (cueing or control) was randomly assigned, and a highly trained physiotherapist recorded the time taken to complete the walking task and the number of FoG episodes, which occurred while the task was performed. The preferred stimulation site (hamstring or quadriceps) was chosen by the participant, and the stimulation intensity level was configured by a physiotherapist at the highest tolerable level such that a sensory response was elicited but that the amplitude was not of sufficient intensity to produce a motor response.

Upon completion of the walking tasks, a participant questionnaire was completed, which incorporated a standard 100 mm Visual Analogue Scale (VAS) and a Face Pain Rating Scale to assess the perceived comfort/pain level of the sES cueing.

### 2.4. Statistical Analysis

Data were analyzed by paired *t*-tests for both the time to complete a walking task and the number of FoG episodes occurring. Analysis was performed with SPSS version 24 (IBM Corporation, NY, USA).

## 3. Results

A total of 64 FoG episodes were identified during “control” and “cueing” conditions. All participants except P9 experienced at least one FoG episode during the control condition. The average time to complete the walking task during control conditions was 99.03 ± 36.12 seconds, while the average time to complete the walking task during cueing conditions was 86.04 ± 37.93 seconds (Table 2). In addition, we observed on average 4.89 ± 3.48 FoG episodes occurring under control conditions and 2.22 ± 2.89 FoG episodes under cueing conditions. The greatest effect of cueing was observed in the participant P4, with a reduction in time taken to complete the walking task and the number of FoG episodes occurring of 32% and 75%, respectively. These data show that sES had a positive effect for all participants with regard to the time taken to complete a walking task. For participants that experienced FoG during the control walking task (*n*=8), the data show that sES also had a positive effect with regard to the number of FoG episodes occurring.

During sES cueing, the mean reduction in time taken to complete the walking task and the occurrence of FoG episodes was 14.23 ± 11.15% and 58.28 ± 33.89%, respectively. There is a significant difference in the time taken to complete the walking task for control and cueing conditions (*t* (8) = 3.46, *p*=0.009). There was also a statistically significant difference in the number of FoG episodes occurring during control and cueing conditions (*t* (8) = 4.619, *p*=0.002).

Despite the positive effects observed, the wider adoption of sES as a cueing strategy by PwP will be dependent not just on the efficacy of the cue but also on the acceptability and usability of the device. Thus immediately after the trial with sES, we assessed the user experience with the device in terms of comfortable and tolerance (100 mm VAS score) and perceived comfort/pain (Face Pain Rating Scale). Participant VAS scores ranged from 5.3 (mildly comfortable) to 9.3 (very comfortable) with a mean score of 7.88 (comfortable) for the perceived comfort of sES cueing. All participants gave the Face Pain Rating Scale score of 0 (no pain), with the exception of P6 who gave a score of 2 (mild, annoying pain) for the pain level of sES cueing.

## 4. Discussion

We investigated the feasibility of using sES cueing as a method of reducing FoG in PwP and consequently improving gait performance. The specific aim of the study was to investigate if a “fixed” rhythmic sES cueing strategy could improve the time taken to complete a walking task and reduce the number of FoG episodes occurring during the performance of the task in a population known to freeze. Our data clearly show that each participant completed their self-defined walking task quicker when sES cueing was applied. We also observed a reduction in the number of FoG episodes occurring during the performance of that walking task with cueing (freezers only, *n*=8).

In 2016, Benoît et al. carried out a preliminary investigation of the immediate effects of sensory ES (sES) cueing with 9 test subjects exhibiting FoG [15]. While the study did not demonstrate a statistically significant effect of sES cueing, there was a clear trend indicating that sES when applied at heel off could reduce both the time to complete a walking task and the number of FoG episodes occurring when performing the task. However, we hypothesized that further positive effects of sES cueing may be achieved by adopting a “fixed” rhythmic sES cueing strategy. We found a 14% reduction in time to complete a walking task and a 58% reduction in the number of FoG episodes occurring. This compares with 19% and 12% reported in a previous sES study [15]. While these studies were not identical and were not carried out on the same population, the results compare favorably.

**Figure 2:**
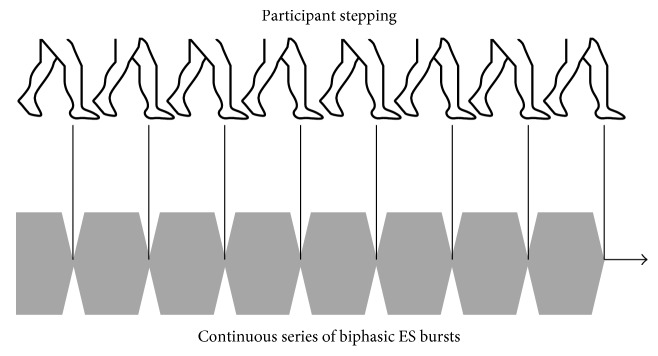
Rhythm of “fixed” sES and synced step rate of participants (700 ms).

A few mechanisms may be responsible for the positive results of the sES cueing strategy we adopted: (i) it may be possible that participants adapted to the cue by synchronizing their step time (557 ms; range: 500 ms and 667 ms) to the “fixed” rhythm of the delivered sES cue (700 ms; Figure 2), (ii) participants may have failed to adapt to the cue and instead artificial stimulation of the proprioceptive pathways may have enhanced proprioceptive information processing as suggested by Pereira et al. [23], or (iii) participants adapted to the cue by synchronizing their step rate and artificial stimulation of the proprioceptive pathways enhanced proprioceptive information processing.

While our data show that sES had a positive effect for all participants with regard to the time taken to complete a walking task (*n*=9) and for all participants who experienced FoG, on the number of FoG episodes occurring (*n*=8). Some participants responded strongly to cueing while others did not experience the similar beneficial effects. Although similar diversity in results has been reported in previous cueing studies, the explanation for this diversity in results is unclear [8, 15, 24] and merits further investigation.

We hypothesized that any improvement in the time taken to complete the walking task during sES cueing would be directly related to the reduction in FoG episodes achieved during completion of that task. In most cases, this was true; the greater the reduction in FoG episodes, the quicker the participant completed the walking task. However, while P9 did not exhibit FoG during any condition, when sES cueing was active, the walking task was completed 25.87% quicker, suggesting that sES cueing impacted positively on gait fluency, even in the absence of FoG.

In addition to the sES study of Benoît et al., our data compare favorably with studies by McAuley et al. [7], where auditory and visual cueing reduced the completion time of a walking task by 12.1% and 5.3%, respectively, and Arias and Javier [8], where auditory cueing reduced the number of FoG episodes by approximately 50% (standard deviation 58.82%) during a walking task.

## 5. Limitations of Study

Our conclusions are limited by the fact that the study population was composed of 9 participants, and during the study, one participant did not experience FoG and another participant only experienced one FoG event. Another limitation and common difficultly in studying FoG is that the presence of a clinician (physiotherapist) may have reduced the likelihood of FoG occurring. Therefore, the results must be interpreted with caution due to the small sample size and variability in the number of FoG episodes per participant. Therefore, while the data indicate a positive effect, a further larger study is required to more comprehensively evaluate the effect of the sES cueing.

## 6. Conclusion

This study, although limited in participant numbers, provides some evidence that sES cueing, delivered in a “fixed” rhythmic manner, may reduce FoG episodes in PwP and enhance walking performance. This preliminary work demonstrated encouraging results and supports the exploration of a larger study to confirm whether or not these findings are reproduced in a larger sample size.

## Figures and Tables

**Table 1:** Walking tasks identified by the participants and common features.

Index	Walking task
P1	Walk from the living room to the kitchen and move the kettle from counter to counter, walk to the front door, open and close the door, and walk to the kitchen then to the front door and finally to the living room.

P2	Walk from the living room to the kitchen sink and then to the bedroom and sit on the bed.

P3	Walk from the living room to the back door, walk through a narrow path to the front door, and finally walk to the living room.

P4	Walk from the living room to the kitchen, walk upstairs into the bedroom, turn at window, and walk downstairs and back to the living room.

P5	Walk from the living room to the toilet, then walk to the bedroom, walk to the kitchen, and finally walk to the living room.

P6	Stand and walk from the living room to the toilet, then sit and stand, walk out to the corridor and turn, and walk back to the living room and sit on chair.

P7	Walk from the living room to the bedroom, then walk to the kitchen, walk to the spare room, and finally return to the living room.

P8	Walk from the living room upstairs into the study room, then walk to the bedroom and bathroom, and walk downstairs and back to the living room.

P9	Walk from the living room to the kitchen, and then walk to the bathroom and back to the living room.

**Table 2:** Participants' results of testing, time to complete task, number of FoG episodes occurring, and percentage of reduction.

	Time (s) to complete task (number of FoG episodes occurring)		Reduction (%) in time to complete task (reduction (%) in the number of FoG episodes occurring)
	Control	Cueing	Cueing
P1	86.18 (8)	79.8 (6)	7.4% (25%)
P2	93.32 (10)	85.36 (6)	8.53% (40%)
P3	66 (3)	59.1 (0)	10.45% (100%)
P4	103.9 (8)	70.2 (2)	32.44% (75%)
P5	112 (4)	81 (1)	27.68% (75%)
P6	86.4 (3)	80.7 (1)	6.60% (66.7%)
P7	113.8 (7)	106.3 (4)	6.59% (42.86%)
P8	178.4 (1)	173.9 (0)	2.52% (100%)
P9	51.26 (0)	38 (0)	25.87% (0%)
Mean (SD)	99.03 ± 36.12 (4.89 ± 3.48)	86.04 ± 37.92 (2.22 ± 2.49)	14.23 ± 11.15% (58.28 ± 33.89%)

## Data Availability

The data used to support the findings of this study are available from the corresponding author upon request.
